# Immobilized NRG1 Accelerates Neural Crest like Cell Differentiation Toward Functional Schwann Cells Through Sustained Erk1/2 Activation and YAP/TAZ Nuclear Translocation

**DOI:** 10.1002/advs.202402607

**Published:** 2024-07-01

**Authors:** Georgios Tseropoulos, Pihu Mehrotra, Ashis Kumer Podder, Emma Wilson, Yali Zhang, Jianmin Wang, Alison Koontz, Nan Papili Gao, Rudiyanto Gunawan, Song Liu, Laura M. Feltri, Marianne E. Bronner, Stelios T. Andreadis

**Affiliations:** ^1^ Department of Chemical and Biological Engineering University at Buffalo Buffalo NY 14260 USA; ^2^ Department of Pharmacy Brac University Dhaka 1212 Bangladesh; ^3^ Hunter James Kelly Research Institute Jacobs School of Medicine and Biomedical Sciences State University of New York at Buffalo Buffalo NY 14203 USA; ^4^ Department of Biochemistry Jacobs School of Medicine and Biomedical Sciences State University of New York at Buffalo Buffalo NY 14203 USA; ^5^ Department of Biostatistics and Bioinformatics Roswell Park Comprehensive Cancer Center Buffalo NY 14203 USA; ^6^ Division of Biology and Biological Engineering California Institute of Technology Pasadena CA 91126 USA; ^7^ Center for Cell Gene and Tissue Engineering (CGTE) University at Buffalo Buffalo NY 14260 USA; ^8^ Department of Neurology Jacobs School of Medicine and Biomedical Sciences State University of New York at Buffalo Buffalo NY 14203 USA; ^9^ Department of Biomedical Engineering University at Buffalo Buffalo NY 14260 USA; ^10^ Center of Excellence in Bioinformatics and Life Sciences Buffalo NY 14203 USA

**Keywords:** chick embryo, demyelinating diseases, neural crest stem cells, neuregulin, schwann cells

## Abstract

Neural Crest cells (NC) are a multipotent cell population that give rise to a multitude of cell types including Schwann cells (SC) in the peripheral nervous system (PNS). Immature SC interact with neuronal axons via the neuregulin 1 (NRG1) ligand present on the neuronal surface and ultimately form the myelin sheath. Multiple attempts to derive functional SC from pluripotent stem cells have met challenges with respect to expression of mature markers and axonal sorting. Here, they hypothesized that sustained signaling from immobilized NRG1 (iNRG1) might enhance the differentiation of NC derived from glabrous neonatal epidermis towards a SC phenotype. Using this strategy, NC derived SC expressed mature markers to similar levels as compared to explanted rat sciatic SC. Signaling studies revealed that sustained NRG1 signaling led to yes‐associated protein 1 (YAP) activation and nuclear translocation. Furthermore, NC derived SC on iNRG1 exhibited mature SC function as they aligned with rat dorsal root ganglia (DRG) neurons in an in vitro coculture model; and most notably, aligned on neuronal axons upon implantation in a chick embryo model in vivo. Taken together their work demonstrated the importance of signaling dynamics in SC differentiation, aiming towards development of drug testing platforms for de‐myelinating disorders.

## Graphical abstract

Altering signaling dynamics by immobilized NRG1 led to YAP/TAZ activation and differentiation of skin derived Neural Crest (NC) like cells toward functional Schwann Cells (SC) that are associated and aligned with neuronal axons in vitro and in vivo. These findings suggest that NC from human epidermis may provide an easily accessible source of functional SC for treatment of neurological disorders.

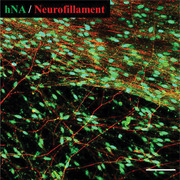

## 1 Introduction

Neural Crest Stem cells (NC) is a transient and migratory population of stem cells that arise from the junction of the neuronal and non‐neuronal ectoderm, namely the Neural Plate Border.^[^ ^1^, ^2^ ^]^ After neurulation, during formation of the neural tube, the neural folds of the ectoderm converge at the dorsal midline. Subsequently NC undergo epithelial to mesenchymal transition (EMT), delaminate from the neuroepithelium and contribute to the formation of the craniofacial skeleton, peripheral neurons and glia, enteric tissue, aortic smooth muscle, skin pigmentation and others.^[^ ^3^ ^]^ Clonal differentiation assays demonstrated the multipotency and self‐renewal potential of NC but also heterogeneity with regards to their differentiation potential.^[^ ^4^ ^]^ Recent studies showed that Schwann Cell Precursors (SCP) are the most multipotent derivative cell type of NC, giving rise to parasympathetic and enteric neurons, pigment cells and importantly, mature Schwann cells, which are responsible for myelination of the peripheral nervous system (PNS) and promote post injury nerve regrowth.^[^ ^5^, ^6^ ^]^ Interestingly, mature Schwann cells of the PNS have the potential to differentiate postnatally into melanocytes, and enteric neurons.^[^ ^6^, ^7^, ^8^ ^]^ The process for Schwann Cell (SC) differentiation is complex, with a variety of signaling pathways affecting fate specification.^[^ ^9^ ^]^ The pivotal step of axonal sorting, when immature Schwann cells choose which neuronal axons to myelinate, is controlled by extracellular signals, such as NRG1‐ERBB2/3, Notch and extracellular matrix, that results in activation of the Hippo‐YAP/TAZ pathway.^[^ ^10^, ^11^ ^]^

It is well established that certain genetic mutations result in abnormal NC and SCP development leading to many congenital diseases, such as cardiovascular defects and craniofacial abnormities, collectively known as neurocristopathies,^[^ ^12^ ^]^ myelopathies and neurodegenerative diseases. Therefore, patient‐specific adult sources of human NC cells may provide a source of stem cells for treatment of neurodegenerative diseases as well as an in vitro model system to study human disease. In this regard, recent studies have successfully isolated NC‐like cells from different adult tissues, including the hair follicle, craniofacial sources such as the palate and the oral mucosa.^[^ ^13^, ^14^, ^15^, ^16^ ^]^ Recently, our laboratory showed that NC‐like cells could be derived from cultures of epidermal keratinocytes (KC) isolated from glabrous neonatal foreskin, without genetic manipulation. KC‐derived NC (KC‐NC) could be coaxed to differentiate into functional NC fates (neurons, Schwann cells, melanocytes, osteocytes, chondrocytes, adipocytes and smooth muscle cells), in vitro and in lineage tracing experiments in chick embryos.^[^ ^16^, ^17^ ^]^ These cells can also be expanded and retained in their multipotent state for up to a month.^[^ ^18^ ^]^ Given the accessibility of human skin, KC‐NC may provide a valuable source of multipotent stem cells for treatment of myelopathies and other debilitating neurodegenerative diseases. Therefore, it is critical to develop robust differentiation protocols leading to stable and sustained SC fate for disease modeling and in vivo cell transplantations. To this end, our lab has recently reported successful differentiation of KC‐NC to SC utilizing a microfiber substrate where NRG1 was conjugated to heparin molecules covalently bound on polydopamine coating.^[^ ^19^ ^]^

Following these pivotal findings, we hypothesized that sustained signaling dynamics through NRG1‐ERBB2/3 interaction may be necessary for successful SC differentiation. To this end, we employed an immobilized fusion protein containing NRG1 fused to an Fc domain (NRG1‐Fc or iNRG1) as a way to affect NRG1 signaling through sustained immobilized NRG1 (iNRG1)‐ERBB2/3 interaction. Indeed, sustained phosphorylation of downstream pathways Erk1/2, Akt and MAP38 led to upregulation of SC mature markers. Interestingly, phospho‐Erk1/2 led to YAP1 nuclear translocation, upregulation of laminin receptors and the mature cytoskeletal glia marker, GFAP. Lastly, the capacity of differentiated SC for axonal alignment was tested in two physiological models: i) in vitro, using co‐cultures with rat DRG neurons; and ii) in vivo, by assessing their migration toward TUJ1+ neurons and alignment along neuronal axons upon implantation into chick embryos.

## 2 Results

### 2.1 Single Cell RNA‐Sequencing of KC‐NC Indicated SCP/SC Differentiation Potential

Previous work from our lab demonstrated the derivation of NC‐like cells from neonatal keratinocytes (KC) that exhibited multipotent differentiation toward classical NC derivatives. During a 7‐day induction with a multi‐growth factor media, KC cluster together, while KC‐NC delaminated out of the KC colony and proliferated, yielding a highly heterogeneous population of KC‐NC. To examine the heterogeneity of KC‐NC and how their transcriptomic profile differs from that of their parental KC, we employed single cell RNA sequencing (scRNA‐Seq). To this end, after isolation from human epidermal keratinocytes from neonatal glabrous foreskin, we performed a 7‐day induction toward KC‐NC as we described previously.^[^ ^16^, ^20^ ^]^ The induction media (FI) contained FGF2, IGF1, Ascorbic Acid, Heparin, Hydrocortisone and 2% FBS. KC from the same donor were grown in Keratinocyte Serum Free Media (KSFM). RNA was isolated from KC, denoted as Day 0 of induction, and from days 2, 4, and 7 of FI induction. RNA from single cells from each of the different time points was barcoded, labeled, and sequenced by the 10x Genomics Chromium platform and the resulting data files were processed with the Bio Turing Browser.

**Figure 1:**
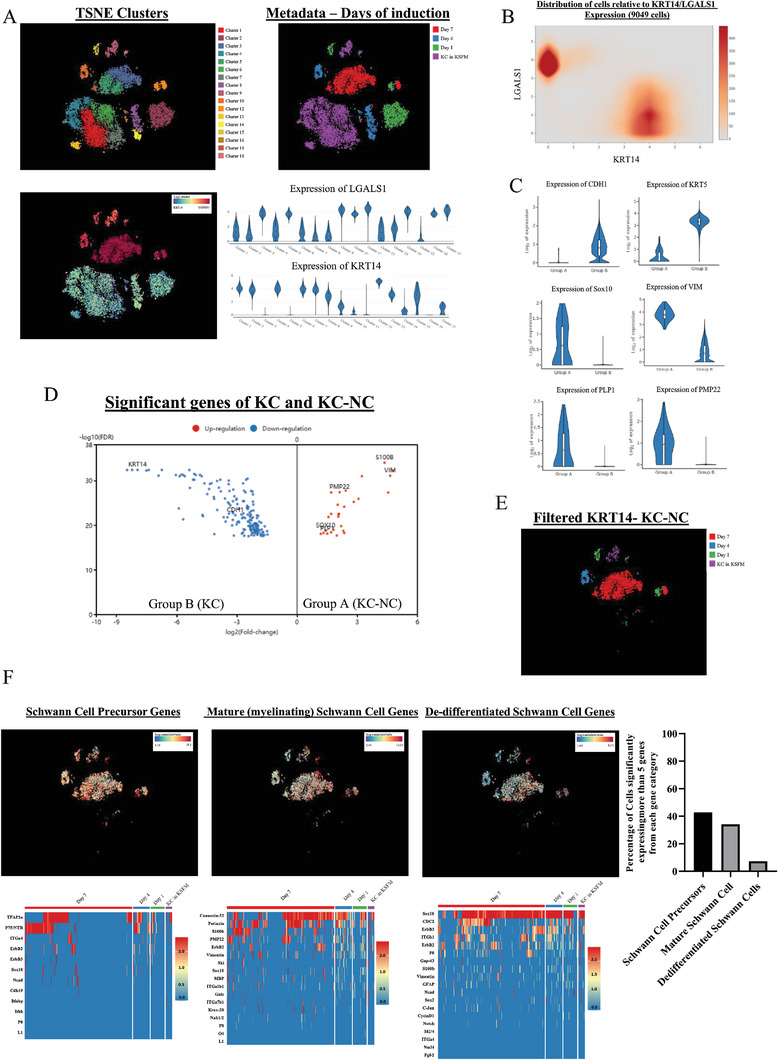
Neural Crest‐like Cells derived from Epidermal Keratinocytes (KC) display differentiation propensity toward Schwann Cells. A) Single cell RNA‐seq displays clustering of KC‐NC away from the K14+ cluster of KC. LGALS1 and K14 separate KC and KC‐NC in two distinct clusters. B) Separation of KC and KC‐NC populations through K14 and LGALS1 genes. Sample was filtered with respect to LGALS high and K14 low cells to isolate the KC‐NC population. C) Volcano plot showing significantly upregulated genes between KC and KC‐NC populations. D) Violin plots indicating the upregulation of key KC and Neural Crest/Pre‐Schwann Cell genes between the KC and the KC‐NC populations. E) Enrichment analysis showing significantly upregulated and downregulated pathways between KC and KC‐NC populations (*p* < 0.001). F) t‐SNE plot and heatmaps of three groups of genes (Schwann Cell Precursors, Mature Schwann Cells, De‐Differentiated Schwann Cells) showing a larger percentage of KC‐NC cells expressing SCP and Mature SC markers than De‐differentiated SC markers.

Single cells from all samples were pooled in the same dataset for analysis, clustered through k‐means clustering and plotted in a t‐SNE plot (**Figure** **1**A). We overlaid the metadata containing information about the experimental time points on the k‐means clusters and interrogated the dataset for the highest differentially expressed genes. We discovered that expression of Galectin 1 (LGALS1) and Keratin 14 (KRT14) distinguished our dataset into two major cell populations (Figure 1B). Violin plots show that the KRT14 high (KRT14^hi^) cells, expressed epidermal genes (CDH1, KRT5) indicating epidermal keratinocytes (Figure 1C); while the LGALS1‐high (LGALS1^hi^) cluster expressed high levels of Schwann Cell and myelin related genes, such as Sox10, S100b, PMP22, and VIM. All the genes that were differentially expressed in the two groups are shown in the volcano plot between groups A (KC‐NC) and B (KC) (Figure 1D).

Next, we filtered out the KRT14^hi^ (Figure 1E) cells and further interrogated the LGALS1^hi^ cells with gene lists containing bona fide genes from the three stages of Schwann cell differentiation cascade: Schwann Cell Precursors (ScP), Myelinating (mature) Schwann cells (MSc) and De‐differentiated Schwann cells (DSc). We observed that our KC‐NC population is quite diverse with 42.6% of cells expressing five or more ScP‐specific genes (P75/NTR, ErbB2/3, Sox10, ITG*α*4, Bfabp); 32.8% expressing five or more myelination or MSc‐related genes (PMP22, Krox20, S100b, Vimentin, Galc) and 7.3% expressing five or more DSc genes (C‐Jun, Gap‐43, ITG*β*1, CDC2, Sox2) (Figure 1F). While diverse, KC‐NC expressed several SC‐specific genes, suggesting that they may have high propensity to differentiate toward mature SC.

### 2.2 Immobilized NRG1 Sustains Erk1/2, Akt and MAP38 Phosphorylation

Our previous work showed that KC‐NC can differentiate to Schwann cells, but differentiation required 4–5 weeks of culture in the appropriate differentiation medium. An important factor that promotes Schwann cell differentiation is NRG1, which binds to Erb2/3 and activates downstream signaling pathways that are critical for Schwann cell specification.^[^ ^21^ ^]^ In vivo binding of Schwann cells to neurons is mediated via NRG1‐Erb2/3 interaction, which activates pathways leading to axonal sorting and myelination.^[^ ^22^ ^]^ Based on this, *we hypothesized that generating a neuron‐mimetic surface might alter the dynamics of cell signaling downstream of the Erb2/3 receptor and promote KC‐NC differentiation toward Schwann cells*.

**Figure 2:**
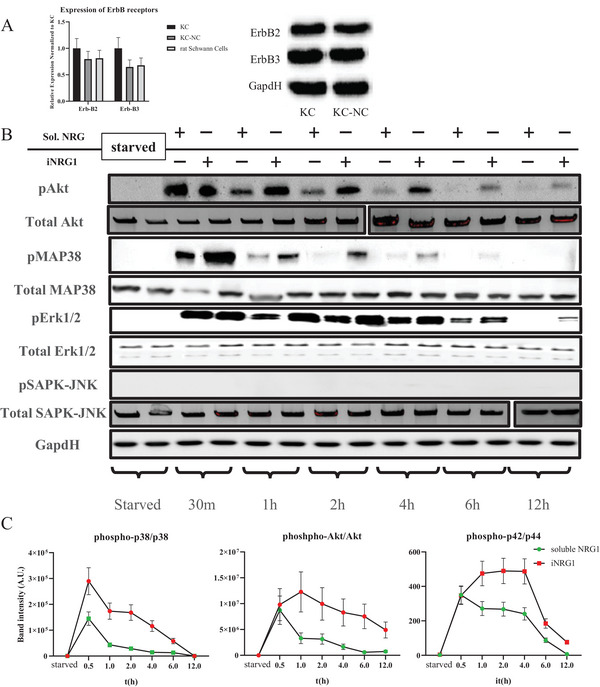
Immobilized NRG1 sustains phosphorylation of downstream NRG1‐ERBB2/3 pathways A) mRNA and proteins levels of ERBB2 and ERBB3 receptors. B) Western blot demonstrating phosphorylation of Akt, Erk1/2, MAP38 and SAPK‐JNK for up to 12 h. C) Quantification of the relative phosphorylation of the indicated kinase normalized by the total amount of each protein. The housekeeping protein, GAPDH served as loading control. All values are mean ± S.D. Each experiment was repeated three times.

To this end, first we measured the expression of ErbB2 and ErbB3 mRNA and protein levels, and observed no significant difference between KC, KC‐NC or rat sciatic SC that served as control (**Figure** **2**A). Subsequently, we employed a fusion protein NRG1‐Fc that was immobilized via the Fc domain (iNRG1) on a non‐tissue culture plate.^[^ ^23^ ^]^ KC‐NC, cultured for 7 days in FI medium were plated on iNRG1 or Collagen 1 (Col1) coated surface, while soluble NRG1 (sNRG1) was provided in the medium. We tested the efficiency of sNRG1 and iNRG1 in a time‐ and dose‐dependent manner through the phosphorylation of ErbB2 receptor. Indeed, both sNRG1 and iNRG1 induced ErbB2 in a dose dependent manner (Figure S4A, Supporting Information) and based on these results, we chose concentrations that saturate the ErbB2 receptor phosphorylation to achieve optimal differentiation. Furthermore, we observed that iNRG1 sustained ErbB2 phosphorylation over 4 h (Figure S4B, Supporting Information). Subsequently, we examined the kinetics of multiple mitogen‐activated protein kinase pathways downstream of the NRG1‐ErbB2/3 interaction by iNRG1 versus sNRG1. Interestingly, immobilized iNRG1 induced sustained phosphorylation of Akt and MAP38, while phosphorylation by sNRG1 was more transient. Notably, Erk1/2 which plays an important role in SC migration and axonal sorting,^[^ ^24^ ^]^ was phosphorylated for up to 12 h on iNRG1. On the other hand, JNK, which has been implicated in SC de‐differentiation^[^ ^25^, ^26^ ^]^ toward a quiescent non‐myelinating phenotype, was not phosphorylated (Figure 2B,C).

### 2.3 iNRG1 Promotes SC Differentiation Faster than sNRG1

**Figure 3:**
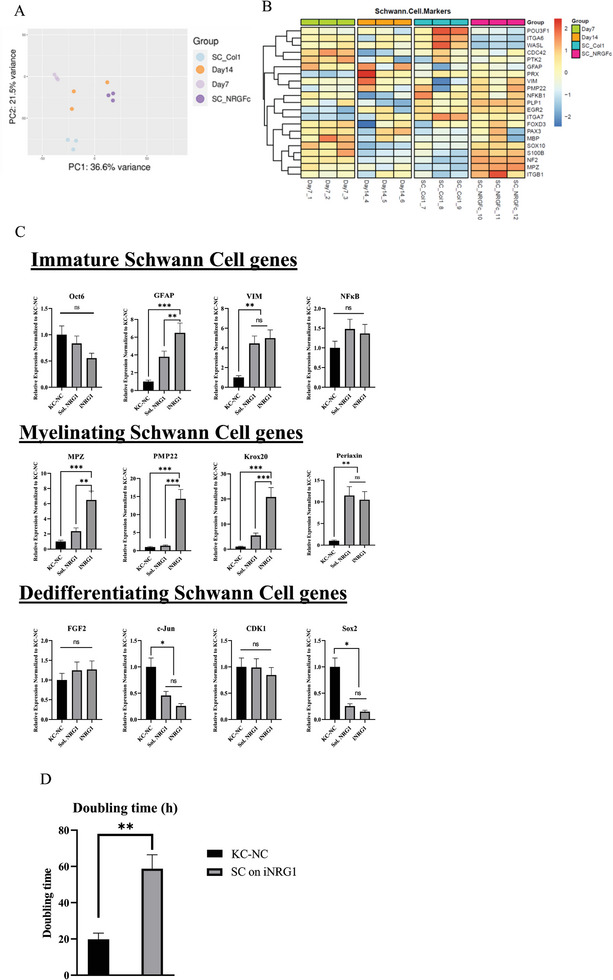
Differentiation on immobilized NRG1 upregulated mature SC markers at the mRNA level. A) PCA plot of the indicated RNA‐seq samples showing that KC‐NC that are differentiated in iNRG1 (SC_NRG‐Fc) are clustered separately from those differentiated with sNRG1 on collagen (SC_Col1) and naïve KC‐NC on day 7 or 14. B) Heatmap of notable SC genes among samples of Day 7 and 14 post KC‐NC induction and differentiation sNRG1 or iNRG1. C) Verification of RNA‐seq by qRT‐PCRs of immature (Pre‐SC), myelinating and de‐differentiating SC genes. D) Doubling times of naive KC‐NC and differentiated to SC on iNRG1.

Next, we differentiated KC‐NC toward SC fate for two weeks, while passing the cells every 3 days on a fresh iNRG1 substrate. Bulk RNA‐seq was performed under four conditions: KC‐NC after 7 days or 14 days of induction; KC‐NC differentiated on a Col1 substrate using sNRG1; and KC‐NC differentiated on iNRG1. Principal component analysis showed that the samples clustered together in distinct groups with cells differentiated under soluble NRG1 or iNRG1 clustering apart from each other and the undifferentiated KC‐NC (**Figure** **3**A). As shown in Figure 3B differentiation on iNRG1 induced significant upregulation of SC mature markers such as MPZ, PMP22, and ERG2 (Krox20). In addition RT‐PCR verified the results showing significant upregulation of mature, myelinating SC genes on iNRG1 as compared to sNRG1; markers of de‐differentiating SC showed no change; while cJun, a negative regulator of myelination,^[^ ^27^ ^]^ was downregulated during differentiation (Figure 3C). Pathway analysis using the top variable genes input, indicated that SC differentiated by iNRG1 upregulated multiple pathways related to neuronal or Schwann cell development and signaling (Figure S5A,B, Supporting Information). Interestingly, differentiation with sNRG1 did not upregulate any pathways related to neuron or Schwann cell function (Figure S5C, Supporting Information). In addition, the doubling time of cells on iNRG increased significantly, indicating differentiation (Figure 3D).

**Figure 4:**
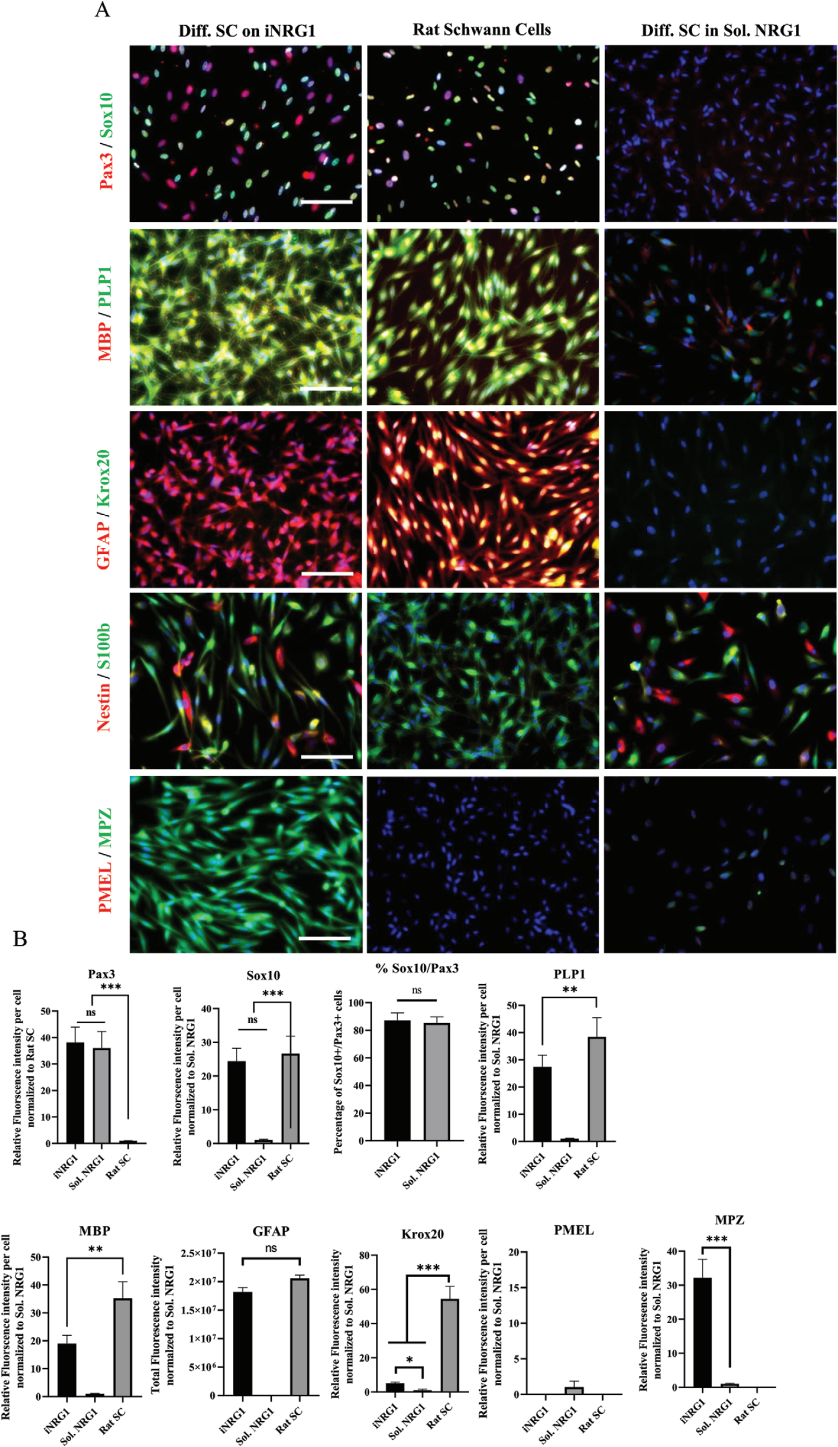
iNRG1 induces SC maturation. A) Immunostaining for SC specific markers between the indicated samples: KC‐NC differentiated to SC on iNRG1, rat SC and KC‐NC differentiated in soluble NRG1. B) Quantification using ImageJ. Scale bars, 50 µm. All values are mean ± S.D. Each experiment was repeated three times.

Immunocytochemistry showed significant upregulation of important mature SC markers, like MPZ, Krox20, PLP1, GFAP and intracellular MBP. The levels of expression were similar to rat sciatic SC explanted from rat DRGs (**Figure** **4**A,B). PMEL was not upregulated, indicating that the differentiation protocol did not promote melanocytic fate, although KC‐NC have the potential to differentiate into melanocytes, as shown previously.^[^ ^16^, ^28^ ^]^ Expression of Sox10, a transcription factor necessary for the upregulation of Krox20 and MPZ—reportedly through binding with HDAC1/2 complex on the Krox20 and MPZ promoter^[^ ^29^, ^30^, ^31^ ^]^ was sustained on iNRG but was downregulated on sNRG1 substrate. These results suggested that iNRG sustained phosphorylation of Erk1/2, Akt or MAP38 pathways and enhanced differentiation to SC, possibly via transcriptional regulation of Sox10.

### 2.4 Sustained iNRG1 Signaling Induced YAP1 Nuclear Translocation via Erk1/2

**Figure 5:**
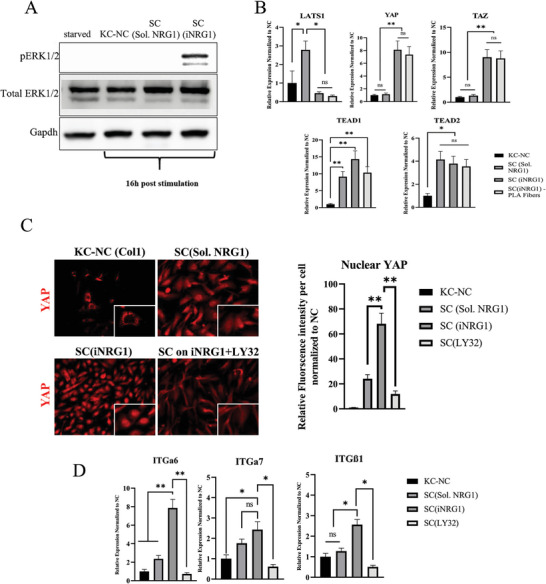
Immobilized NRG1 enhances Erk1/2 mediated YAP1 nuclear translocation and integrin expression. A) Western blot demonstrates sustained phosphorylation of Erk1/2 on iNRG1 as compared to soluble NRG1. B) qRT‐PCR for Hippo pathway effectors *Lats1, Yap, Taz*, and *Tead1/2*. C) Immunostaining for YAP1 localization in KC‐NC, SC on Collagen 1 or iNRG1 and during Erk1/2 inhibition by LY3214996 and quantification of nuclear YAP. D) qRT‐PCR for integrin subunits *α*6, *α*7, and *β*1 for the conditions mentioned above. Scale bars, 50 µm. All values are mean ± S.D. Each experiment was repeated three times.

In order to investigate the connection between the phosphorylation of NRG1‐ErbB2/3 downstream pathways and enhanced SC differentiation, we investigated the effects of iNRG1 on the Hippo pathway effectors, Yap/Taz that reportedly play an important role in initiating SC differentiation.^[^ ^32^, ^33^ ^]^ The sustained phosphorylation of Erk1/2 upregulated mRNA expression of Yap and Taz and downregulated expression of LATS1/2 (**Figure** **5**A,B), a complex responsible for Yap phosphorylation, translocation to the cytoplasm and ubiquitination. Interestingly, iNRG1 increased nuclear Yap protein, while chemical inhibition of Erk1/2 by LY3214996 significantly reduced nuclear Yap and diminished expression of integrins *α*6 and *β*1, which are necessary for SC maturation^[^ ^34^ ^]^ (Figure 5C,D). These results suggested that iNRG1 in addition to SC differentiation might also promote cytoskeletal re‐organization processes that are necessary for axonal sorting.

**Figure 6:**
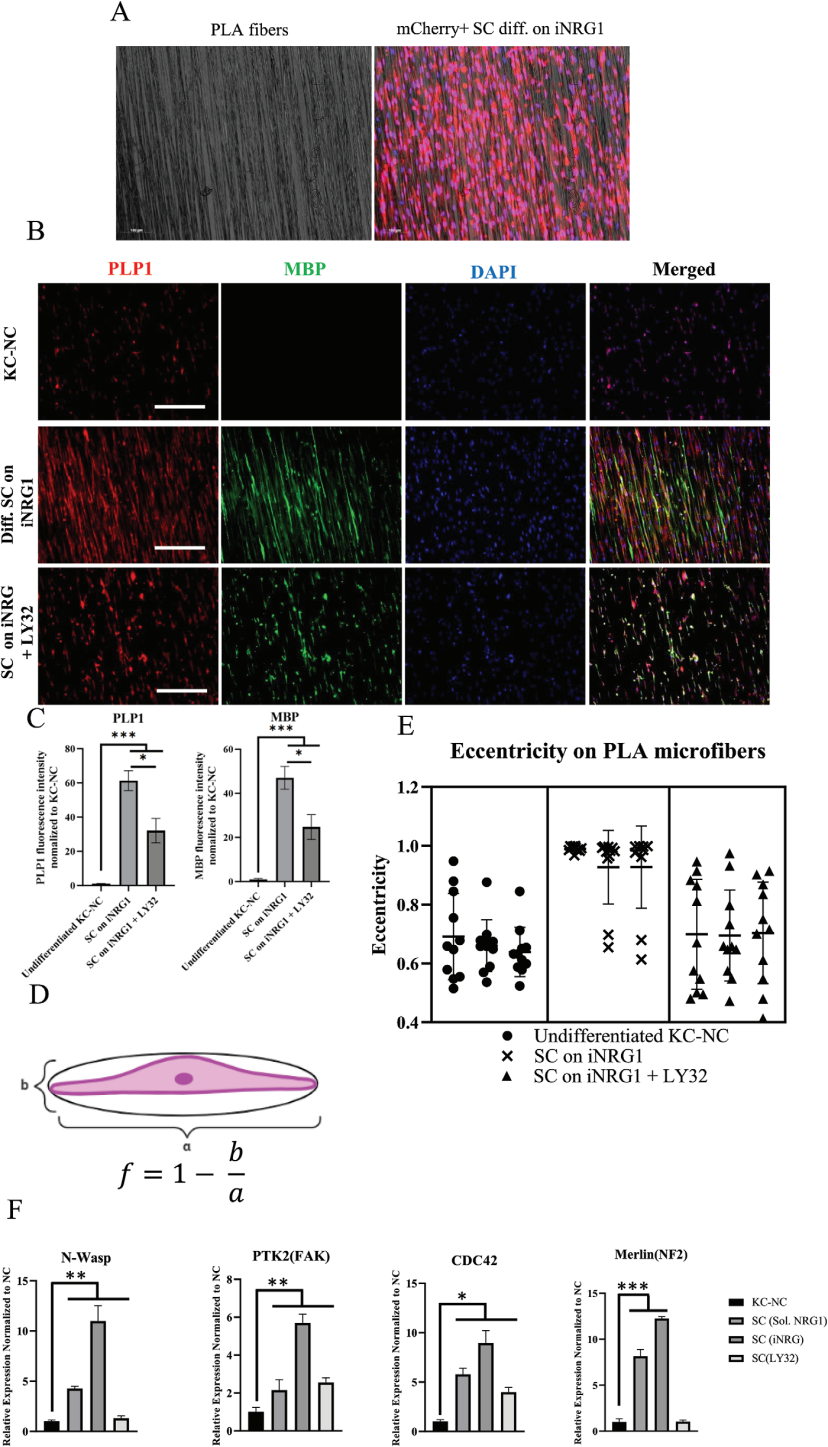
iNRG1 mediated Erk1/2 activation is necessary for alignment on laminin coated PLA fibers. A) Phase image of PLA fibers and mCherry+ SC indicating alignment. B) Immunostaining for PLP1 and MBP on PLA fibers comparing alignment between KC‐NC, SC on iNRG1 coated fibers without or with Erk1/2 inhibition. C) Immunostaining quantification of PLP1 and MBP. D,E) Eccentricity quantification of ICC using Image J. F) qRT‐PCR for the indicated Hippo pathway effectors affecting cytoskeletal reformation. Scale bars, 50 µm. All values are mean ± S.D. Each experiment was repeated three times.

To test this hypothesis, we emulated the neuronal microenvironment with PLA microfibers (diameter of 4–8 µm) that were coated with poly‐L‐ornithine/Laminin and evaluated adhesion and spreading of SC derived from KC‐NC on iNRG1 or undifferentiated KC‐NC. Indeed, spreading of SC on PLA microfibers was significantly enhanced as compared to parental KC‐NC (**Figure** **6**A,B). Interestingly, inhibition of Erk1/2 not only reduced the attachment and spreading of differentiated SC, but also their MBP and PLP1 expression per cell (Figure 6B,C). The cell spreading on the PLA microfibers was calculated by modeling the cells as “ellipsoids” and the degree of their spreading was quantified with respect to how close their eccentricity is to 1, following the formula below,

(1) $$f=1-\frac{b}{a}$$

where b denotes the small and *α* the large diameter. Cell area, large and small diameters were measured using Image J (Figure 6D,E). Furthermore, RT‐PCR indicated that Hippo related genes, such as N‐Wasp, PTK2, CDC42 and Merlin were upregulated by iNRG1 and decreased significantly upon Erk1/2 inhibition (Figure 6F), signifying significant changes of cytoskeletal related genes by iNRG1‐induced sustained activation of Erk1/2.

### 2.5 Co‐Culture with rat DRG Neurons Exhibits Axonal Sorting Potential

These results prompted us to investigate the neuronal alignment and axonal sorting capabilities of KC‐NC derived differentiated SC on iNRG1 substrate using a physiologically relevant system, a co‐culture with rat DRG neurons.^[^ ^11^ ^]^ Dorsal root ganglia were isolated from the spinal cord of rat P13‐P14 embryos and plated in Matrigel on PLL coated glass coverslips. Non‐neuronal cells were eliminated through two cycles of fluoroxidine treatment and rat sciatic SC, or KC‐NC derived SC differentiated on Collagen 1 or iNRG1 were plated on top of the DRG neurites.

**Figure 7:**
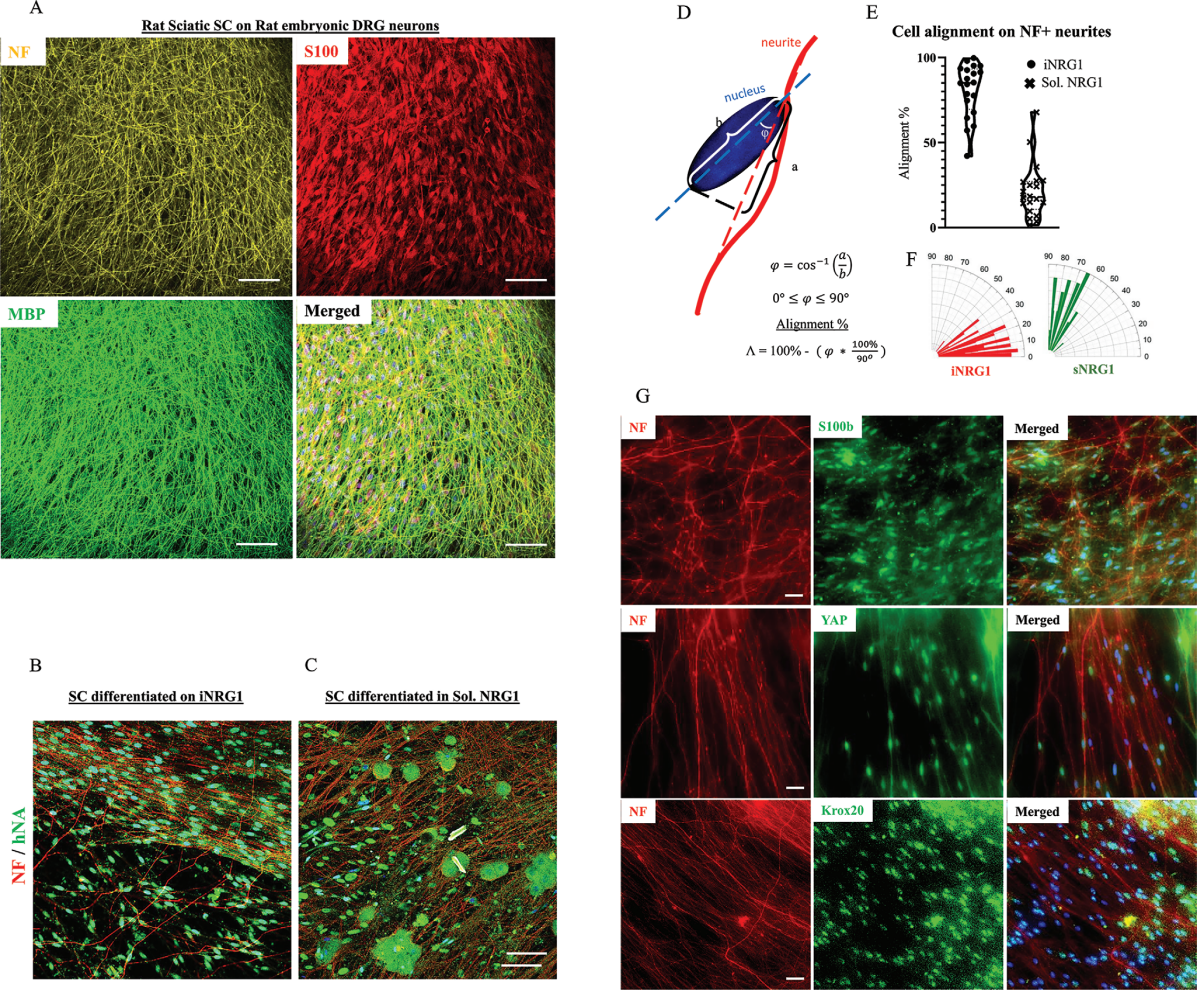
KC‐NC differentiated toward SC on iNRG1exhibit axonal sorting potential on rat DRG neurons. A) Immunohistochemistry rat DRG neurons for NF, and rat sciatic SC for S100b and MBP. B,C) Alignment of immunolabeled hNA+ iNRG1 differentiated SC (B) or naïve KC‐NC (C) on NF+ rat DRG neurons. D–F) Quantification of alignment of KC‐NC and iNRG1 differentiated SC that were placed on rat DRG neurons. G) Immunohistochemistry of NF+ neurons and SC differentiated on iNRG1 for SC specific markers S100b, Krox20 and YAP. Scale bars, 50 µm. All values are mean ± S.D. Each experiment was repeated three times.

After two weeks in ascorbic acid rich media, the rat sciatic SCs aligned along the DRG neurons, as shown by localization of S100b+ Schwann cells and the Neurofillament+ (NF+) neuronal axons (**Figure** **7**A; Figure S1 and Movie S1, Supporting Information). Similarly, human nuclear antigen (hNA)+ SC differentiated on iNRG1 showed alignment with NF+ DRG neurons (Figure 7B; Figure S1 and Movie S2, Supporting Information), demonstrating development of mature SC specific function. Interestingly, iNRG1 differentiated cells were able to align with NF+ neurites with higher efficiency than the ones differentiated in sNRG1 (Figure 7C; Figure S1 and Movie S3, Supporting Information). Furthermore, the difference in alignment potential is evident by the quantification of the angle between the longitudinal midline radius of the human nuclei and the NF+ neurite axons (Figure 7D–F). Notably, the hNA+ cells differentiated on iNRG1 maintain the expression of SC mature markers S100b and Krox20 and localize YAP1 primarily in the nucleus (Figure 7G). This data demonstrates clearly that sustained engagement of the ErbB2/3 receptor not only promotes SC differentiation as evidenced by expression of mature markers and expression of morphogenic signals (NF2/Merlin, N‐Wasp), but also enhances the ability of the differentiated cells to sort axons and align on the neurites, suggesting generation of functional SC.

### 2.6 SC Differentiated on iNRG1 Migrate and Localize on Neuronal Axons In Ovo

**Figure 8:**
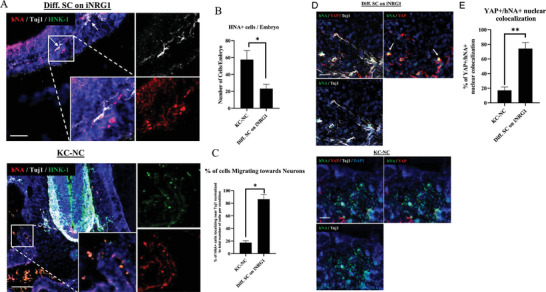
KC‐NC differentiated toward SC on iNRG1 exhibit axonal sorting in ovo. A) Immunostaining for HNK1 and hNA+ KC‐NC and SC differentiated on iNRG1 localization with respect to TUJ1+ neurons in ovo. B) Quantification of total hNA+ cells per embryo and percentage of hNA+ cells localized on TUJ1+ neuronal axons. C) Quantification of the percentage of hNA+ cells migrating towards neurons. D) Immunostaining for YAP localization on hNA+ KC‐NC and SC differentiated on iNRG1 on TUJ1+ neurons in ovo. E) Quantification of the percentage of hNA+ cells, localizing YAP to the nucleous. Scale bars, 100 µm. All values are mean ± SD. Each experiment was repeated three times.

To further demonstrate generation of functional SC, we performed transplantation of undifferentiated KC‐NC or KC‐NC that were differentiated to SC on iNRG1 into chick embryos, and examined their potential to engage in axonal sorting in ovo. To this end, cells were transplanted into the head mesenchyme of 8–13 host chick embryos and were analyzed 72 h post‐transplantation (KC‐NC n = 12, SC n = 16). The transplanted human cells were distinguished from chick cells by labeling with hNA and examined their localization in reference to TUJ1+ neurons. We observed a significant difference in localization of the two cell groups with respect to neuronal axons (**Figure** **8**A), despite the overall higher cell number of hNA+ KC‐NC (Figure 8B). Specifically, 83.4% ± 17.2 of hNA+ iNRG1 derived SC were co‐localized with TUJ1+ neurons, as compared to only 19.1% ± 8.5 of KC‐NC (Figure 8C). The remaining hNA+ undifferentiated KC‐NC were localized in the embryo mesenchyme or the neural tube (Figure S2, Supporting Information). Furthermore, to verify that KC‐NC and SC maintained their phenotype in ovo, we performed histology for HNK‐1 (hybridoma antibody, prepared in the lab of M. Bronner), a marker of migrating NC. Interestingly, HNK‐1 was expressed by undifferentiated KC‐NC, but not SC differentiated on iNRG1 (Figure 8A). Additionally, to verify that the effects of iNRG1 mediated YAP nuclear translocation in vitro persist in ovo, YAP expression by the hNA+ cells among KC‐NC and iNRG1 conditions was measured histologically (Figure 8D). Colocalization analysis between the nuclear expression of hNA and YAP, indicated that cells differentiated on iNRG1 localize YAP preferentially in the cell nucleus compared to undifferentiated KC‐NC (76.3% compared to 38.6%, Figure 8E). Taken together this data demonstrates that SC differentiated on iNRG1 exhibit YAP nuclear translocation and the potential for axonal sorting on TUJ1+ neurons to a higher extent that their KC‐NC precursors, indicating attainment of more mature SC phenotype.

## 3 Discussion

**Figure 9:**
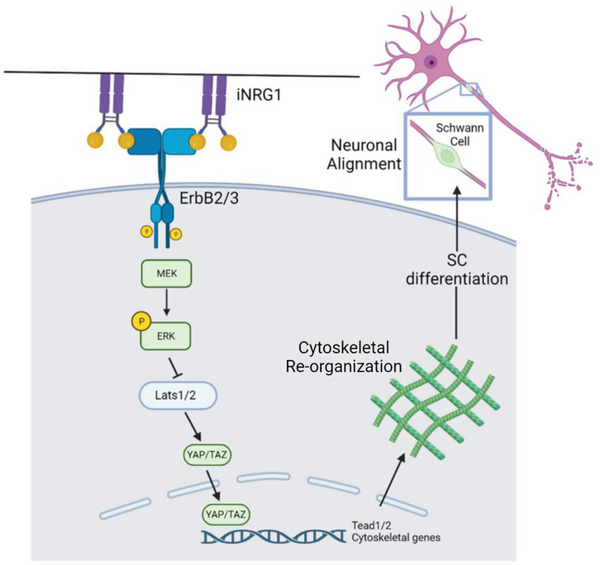
Schematic indicating the potential mechanism of KC‐NC differentiation into SC. Sustained Erk1/2 phosphorylation by the iNRG1–ERBB2/3 interaction leads to YAP/TAZ nuclear translocation with subsequent activation of downstream effectors that lead to SC maturation and enhanced potential to migrate to and align with neuronal axons.

In vitro disease models for neural degenerative disorders as well as stem cell transplantation therapies require a readily available and sustainable cell source, which at the same time, recapitulates the in vivo cellular phenotype accurately. While several protocols for differentiation toward SC fate from various NC sources have been reported previously,^[^ ^35^, ^36^, ^37^, ^38^, ^39^ ^]^ reproducibility and robustness is often limited, due to the variability of the stem cells of origin. Here, we present a differentiation strategy that makes use of a substrate with immobilized NRG1. Binding of KC‐NC on immobilized NRG1 leads to sustained phosphorylation of the ERBB2/3 dimer receptor, promoting differentiation toward SC fate, possibly through YAP nuclear translocation (**Figure** **9**). The Hippo‐YAP/TAZ pathway is reportedly necessary for Schwann Cell myelination, and YAP/TAZ ablation in knockout mice leads to myelin deficiency, thinner myelin sheath and neuronal axon regeneration post injury.^[^ ^40^, ^41^, ^42^ ^]^ YAP/TAZ controls not only cytoskeletal changes and laminin receptors in SC,^[^ ^11^ ^]^ but also the expression proteins necessary for SC maturation and myelination, such as PMP22.^[^ ^43^ ^]^

KC‐NC derived SC on iNRG1 express bona fide markers for myelinating SC such as PLP1, MPZ, Krox20 and GFAP, which are maintained at high levels due to the sustained phosphorylation of ERBB2/3 downstream effectors. Importantly, SOX10 expression is maintained and localized in the nucleus throughout the 2‐week differentiation protocol. SOX10 is a master regulator of neural crest lineage and is necessary for SC fate specification, which requires binding of the HDAC1/2‐PAX3‐SOX10 complex to the promoters of MBP, P0 and Krox20.^[^ ^30^ ^]^ By contrast, SOX10 expression must be shut off to enable NC differentiation toward the neuronal lineages.^[^ ^44^ ^]^ Importantly, ErbB2/3 sustained phosphorylation by iNRG1 does not phosphorylate cJun, a Krox20 antagonist and negative regulator of the myelination process,^[^ ^27^, ^45^, ^46^ ^]^ which is upregulated when the SC injury repair program is activated. Transcriptionally, our differentiated SC express myelination markers, while downregulating de‐differentiation SC effectors.

Admittedly, the field has had little success in human SC myelination,^[^ ^47^ ^]^ and while in vitro oligodendrocyte myelination of DRG neurons has been reported, myelination by human Schwann cells has not been possible to date.^[^ ^48^ ^]^ Notably, iNRG1 derived SC showed much higher potential to align on rat DRG neurons than SC derived on collagen using soluble NRG1. iNRG1 derived SC showed significantly upregulated Merlin/NF2 and integrin genes, suggesting that they may be contributing to enhanced axonal sorting possibly by activating Cdc42 and Rac1.^[^ ^49^, ^50^ ^]^ While the exact mechanism is not known, inhibiting Erk1/2 also inhibited expression of all axonal sorting mediators and abolished SC alignment on PLA fibers or DRG neurons, clearly placing Erk1/2 as a key regulator of the mature SC phenotype, possibly through YAP nuclear translocation. Furthermore, YAP proved to be a key regulator of in ovo SC alignment with chicken neuronal axons, in agreement with previous studies performed in mice.^[^ ^11^ ^]^ Overall, our results suggest that sustained phosphorylation of Erk1/2 may lead to activation of downstream transcription factors above a certain threshold that may be required for successful SC maturation.

Previously, our group showed that KC‐NC isolated from the epidermis can be coaxed to differentiate into all traditional NC derivatives, including SC, neurons, melanocytes and smooth muscle cells,^[^ ^16^, ^17^, ^19^ ^]^ thereby suggesting that they may be an abundant and readily available source for cell transplantation and disease modeling. Our method promotes successful SC differentiation of KC‐NC like stem cells in two weeks without genetic manipulation^[^ ^51^ ^]^ or laborious and time consuming 3D organoid cultures.^[^ ^52^ ^]^ Collectively, our data suggests that immobilized growth/differentiation factors enhance NC differentiation and functionality and may be more broadly applicable to other cell types, e.g., oligodendrocytes, melanocytes, SMC, providing a platform for drug testing and cell therapy for demyelinating diseases.

## 4 Experimental Section

### Isolation of Epidermal Cells

Glabrous (lacking hair follicles) foreskin from 1–3‐day old neonates was procured from John R. Oishei Children's Hospital, Buffalo, NY. Skin samples were washed three times with PBS, dissected into pieces (≈3 cm × 1 cm), enzymatically digested with dispase II protease (Sigma, St. Louis, MO) for 15–20 h at 4 ˚C. The epidermis was then separated from the dermis manually using fine forceps. The separated epidermis was treated with Trypsin‐EDTA (0.25%) (Life Technologies, Carlsbad, CA) for 10–15 min at 37 ˚C, filtered through 70 µm cell strainer (BD Biosciences, Franklin Lakes, NJ), centrifuged and plated on a confluent monolayer of growth‐arrested 3T3‐J2 mouse fibroblast feeder cells in keratinocyte growth medium (KCM) consisting of a 3:1 mixture of DMEM (high glucose) and Ham's F‐12 medium (Life Technologies) supplemented with 10% (v/v) fetal bovine serum (FBS, Atlanta Biologicals, Flowery Branch, GA), 100 nm cholera toxin (Vibrio Cholerae, Type Inaba 569 B, Millipore, Burlington MA), 5 µg mL^−1^ transferrin (Life Technologies), 0.4 µg mL^−1^ hydrocortisone (Sigma), 0.13 U mL^−1^ insulin (Sigma), 1.4 × 10^−4^ m adenine (Sigma–Aldrich), 2 × 10^−9^ m triiodo‐L‐thyronine thyronine (Sigma), 1x antibiotic‐antimycotic (Life Technologies) and 10 ng mL^−1^ epidermal growth factor (EGF, BD Biosciences). The cells were cultured in KCM for 8–10 days. Afterwards, the 3T3‐J2 feeder layer was detached after a 10‐min versene treatment. The remaining cells were treated with trypsin‐EDTA (0.25%), which was then neutralized by a solution containing 10% FBS in PBS and plated in keratinocyte serum free growth medium (KSFM, Epilife medium with Human Keratinocyte Growth Supplement, Life Technologies). Further expansion took place in KSFM prior to NC induction. Passage 1–3 KC were used in all experiments.

### Induction of KC into Neural Crest Stem Cell Fate

For induction into the NC fate, KC were cultured at a density of 8–10 × 10^3^ cells cm^−2^ in collagen type I coated dishes (10 µg collagen type I per cm^2^; BD Biosciences) in the presence of neural crest induction medium (NCIM), comprising of basal medium (EBM‐2 medium; Lonza, Basel, Switzerland) plus 2% (v/v) FBS, 10 µg mL^−1^ heparin (Lonza), 100 µg mL^−1^ ascorbic acid (Lonza) 0.5  µg mL^−1^ hydrocortisone, 1x Gentamicin/Amphotericin‐B (Lonza) and supplemented with 10 ng/ml fibroblast growth factor 2 (FGF2, BD Biosciences) and 10 ng mL^−1^ Insulin like growth factor 1 (IGF1, Lonza). Concentrations FGF2 and IGF1 were optimized in previous studies in our lab.^[^ ^16^ ^]^

### Single Cell RNA Library Preparation and Sequencing

Human interfollicular keratinocytes (KC) and induced KC‐NC after 1, 4, and 7 days were delivered to the UB Genomics and Bioinformatics Core (UBGBC). Cells were counted using the Logos Biosystems LUNA II cell counter with 0.4% Trypan Blue for cell viability. Subsequently, the cells were diluted to 700–1000 cells µl^−1^ in condition media or 1X PBS containing 0.04% BSA. Once diluted, ≈5000 cells were captured on the 10X Genomics Chromium platform using the 3′ transcriptome protocol (V3). The efficient synthesis of cDNA was confirmed, and the samples were processed for Illumina sequencing and quality checked using the Agilent Fragment Analyzer and Qubit fluorescence (Invitrogen). The final concentration of the libraries was measured at 10 nm using the Kapa Biosystems Universal qPCR system. Pooled libraries were diluted and denatured to 250 pM and run on the NovaSeq 6000 SP flow cell (28 × 91).

### Single Cell RNA seq – Bioinformatics Analysis

The sequenced outputs were instated into the 10X Genomics Cellranger v3.0.1 pipeline and subsequently input into the R analysis package Seurat76. Cells with high unique feature counts, high mitochondrial transcript counts, and high ribosomal transcript counts were filtered from the analysis and total cell number as well of mean reads per cell were measured (Figure S3, Supporting Information). The data was normalized using Seurat's LogNormalize, with a scale factor of 10 000. All four datasets were integrated using Seurat's FindIntegrationAnchors followed by IntegrateData and the subsequent DESeq files were uploaded to Bioturing's Bbrowser. K‐means clustering was performed to the combined (pooled) integrated datasets and the metadata were overlayed with the clusters on t‐SNE plots. Utilizing the Bbrowser tool for Differentially Expressed Genes, violin plots and heat maps of characteristic genetic markers were generated.

### Bulk RNA Sequencing and Analysis

The global gene expression profiles of KC‐NC after 7 and 14 days of induction, as well as differentiation through soluble NRG1 and iNRG1 were characterized by next generation RNA sequencing using Illumina platform. Total RNA was isolated for all conditions for three donors using RNeasy Mini Kit and quality control analysis was performed by RNA gel and Agilent Fragment Analyzer. Subsequently, sequencing libraries were prepared as per standard Illumina protocols (Illumina Stranded Total RNA Prep with Ribo‐Zero Plus), quality checked, and quantified by Kapa Biosystems qPCR. Sequencing of the multiplexed libraries was performed in pair‐end (2 × 50 bp) on the NovaSeq 6000 at 300 pM with 1% loading control. Quality control was performed by sequencing reads passed quality filter from Illumina RTA were first processed using FASTQC (v0.10.1). Sample reads were aligned to the human reference genome (GRCh38) and GENCODE (version 38) annotation database using STAR2.17. Gene level raw counts were obtained using Subread19 package. Differential gene expression analysis was performed using R script, where it normalized the gene count data and pre‐filtered low count genes. Pathway analysis was performed utilizing the online GO tool for pathway analysis and was run against MsigDB, a collection of annotated and curated gene set repositories offered by the developer of GSEA (Broad Institute MIT and Harvard). This run used C2 of version 7.4 collection, containing 2307 gene sets from various well‐known and up‐to‐date pathway databases such as BioCarta, KEGG, and Reactome among others.

### Differentiation Toward SC Fate

Non‐tissue culture plates with a hydrophobic surface were incubated overnight at 4 °C with NRG1‐Fc fusion protein (Sino Biological, Beijing China, 100 µg mL^−1^) in phosphate‐buffered saline (PBS) containing 0.9 mm CaCl_2_ and 0.9 mm MgCl_2_. Unbound NRG1‐Fc was washed away and cells were plated for various experiments. KC‐NC or KC cells were plated the next day in KSFM or NCIM and after attachment the media was changed to SC differentiation media containing 10 to 50 ng mL^−1^ NRG1, increasing through 2 weeks of differentiation (Sigma, St. Louis, MO), B27 (Thermo Fisher, Waltham, MA), 2 µm Forskolin for the first 2 days of differentiation (Sigma, St. Louis, MO), 10 ng ml^−1^ BDNF (Thermo Fisher, Waltham, MA) and 1x Glutamax (Thermo Fisher, Waltham, MA). The cells were passed every 3 days on a fresh iNRG1 substrate (two passages over a 15‐day differentiation).

### Cell Culture of Sciatic SC and DRG Neurons

Sciatic rat Schwann cells were produced as described previously^[^ ^53^ ^]^ and cultured in DMEM supplemented with 4.5 g L^−1^ glucose, L‐glutamine, sodium pyruvate, 5% bovine growth serum, penicillin, streptomycin, 0.2% bovine pituitary extract, and 2 µm forskolin. Isolated primary sciatic Schwann cells were used at the third passage. Rat DRG neurons were isolated from E14.5 embryos and established on collagen‐coated glass coverslips as described previously.^[^ ^54^ ^]^ Explants were treated with fluoroxidine (FUDR, Sigma–Aldrich, two cycles) to eliminate all non‐neuronal cells. Neuronal medium was supplemented with 50 ng mL^−1^ NGF (Harlan, Bioproducts for Science). Rat Schwann cells were added (50 000 or 200 000 cells per cover slip) to establish myelinating cocultures of DRG neurons, and myelination was initiated by supplementing the medium with 50 µg mL^−1^ ascorbic acid (Sigma–Aldrich).

### Immunocytochemistry

Cells were washed with cold PBS (4 °C) and permeabilized with 4% (v/v) paraformaldehyde (10 min, 4 °C; Sigma). Permeabilization (10 min, room temperature) was performed with 0.1% (v/v) triton X‐100, (Sigma) in PBS and samples were blocked with 5% (v/v) normal goat serum (Life Technologies) in PBS. The cells were incubated with primary antibodies overnight (4 °C) (Table S2, Supporting Information) followed by incubation with appropriate secondary antibodies (1 h, room temperature) conjugated with Alexa 488 or Alexa 594. Hoechst 33342 (Thermo Fisher Scientific, Grand Island, NY) was used for nuclear staining. Cells that were incubated with only secondary antibody served as controls.

### Quantitative Real Time PCR

Cells were seeded at a density of 10 000 cells cm^−2^ on 6‐well plates coated with iNRG1 or Col1(with soluble NRG1). Undifferentiated cells were used as control. After 14 days of differentiation, total RNA was isolated using RNeasy Mini Kit (Qiagen, Valencia, CA) as per manufacturer's specified protocol. Superscript III cDNA Synthesis Kit (Invitrogen, Waltham, MA) was used to obtain cDNA from 1 µg of isolated RNA per sample. To assess gene expression, real‐time PCR was performed using SYBR Select Master Mix (Applied Biosystems, Waltham, MA) using the primer pairs listed in Table S1 (Supporting Information). For the isolation of mRNA from fibers (Figure 6), the cells were lysed on the fibrous plate as described above.

### Western Blot Analysis

For Western Blot analysis, cells from different conditions were lysed on ice at the timepoints mentioned and the lysate was centrifuged at 15000 x g for 10 min at 4 °C. Supernatants were collected, and the total amount of protein in each sample was determined by Bradford assay. Subsequently, 10 mg of total protein was loaded onto 4% to 20% Tris‐Glycine SDS‐PAGE midi or mini gels. After electrophoresis, proteins were transferred to PVDF membrane (BioRad Laboratories, Hercules, CA) and the expression level of the indicated proteins was then detected using the antibodies. The primary antibodies and antibody dilutions are listed in Table S2 (Supporting Information). ChemiDoc MP imaging system (Bio‐rad) was then used to visualize protein bands and the images were analyzed using the Image Lab software (Bio‐Rad).

### Imaging and Image analysis

Immunocytochemistry images were acquired using a Zeiss Axio Observer Z1 inverted microscope with an ORCA‐ER CCD camera (Hamamatsu, Japan). The images were acquired using fixed exposure time for each fluorescent dye. Cell number quantification was performed using NIH ImageJ. The images were converted to 8‐bit. Manual marking and cell counting was performed for NES+, SOX10+ and FOXD3+ cells using the Cell Counter plugin. For each condition n = 3 separate wells were counted. Statistical significance between the groups was analyzed through one or two‐way ANOVA as indicated and a confidence interval of 95% was chosen. Confocal images were collected by a white light laser scanning Leica Stellaris 5 (Leicamicrosystems, Wetzlar, Germany) with a 20× objective, and the z‐stack depth was within 10 µm (step = 1 µm) and movies were generated using the Leica 3D reconstruction tool. Quantification of cell number and fluorescence intensity was measured using the ImageJ software.

## Conflict of Interest

The authors declare no conflict of interest.

## Supporting information

Supporting Information

Supplemental Movie 1

Supplemental Movie 2

Supplemental Movie 3

## Data Availability

The data that support the findings of this study are available from the corresponding author upon reasonable request.
