# Supplementary material for: Immobilized NRG1 Accelerates Neural Crest like Cell Differentiation Toward Functional Schwann Cells Through Sustained Erk1/2 Activation and YAP/TAZ Nuclear Translocation
Source: Adv Sci (Weinh). 2024 Jul 1;11(33):2402607. doi: 10.1002/advs.202402607 (PMC11633358; doi:10.1002/advs.202402607)
Supplement: Supplementary file 1 — Supporting Information [file ADVS-11-2402607-s001.pdf]

ADVANCED SCIENCE
Open Access

Supporting Information

for *Adv. Sci.*, DOI 10.1002/advs.202402607

Immobilized NRG1 Accelerates Neural Crest like Cell Differentiation Toward Functional Schwann Cells Through Sustained Erk1/2 Activation and YAP/TAZ Nuclear Translocation

*Georgios Tseropoulos, Pihu Mehrotra, Ashis Kumer Podder, Emma Wilson, Yali Zhang, Jianmin Wang, Alison Koontz, Nan Papili Gao, Rudiyanto Gunawan, Song Liu, Laura M. Feltri, Marianne E. Bronner and Stelios T. Andreadis**

Figure S1: 3D reconstruction movies of co-culture with rat DRG neurons. Movie 1: Positive control of rat sciatic SC on DRG neurons. Movie 2: hNA+ SC differentiated with sNRG1 with limited alignment on rat DRG neurons. Movie 3: hNA+ SC differentiated on iNRG1 aligning with DRG neuronal axons.

Movie 1

Rat sciatic SC on rat DRG neurons

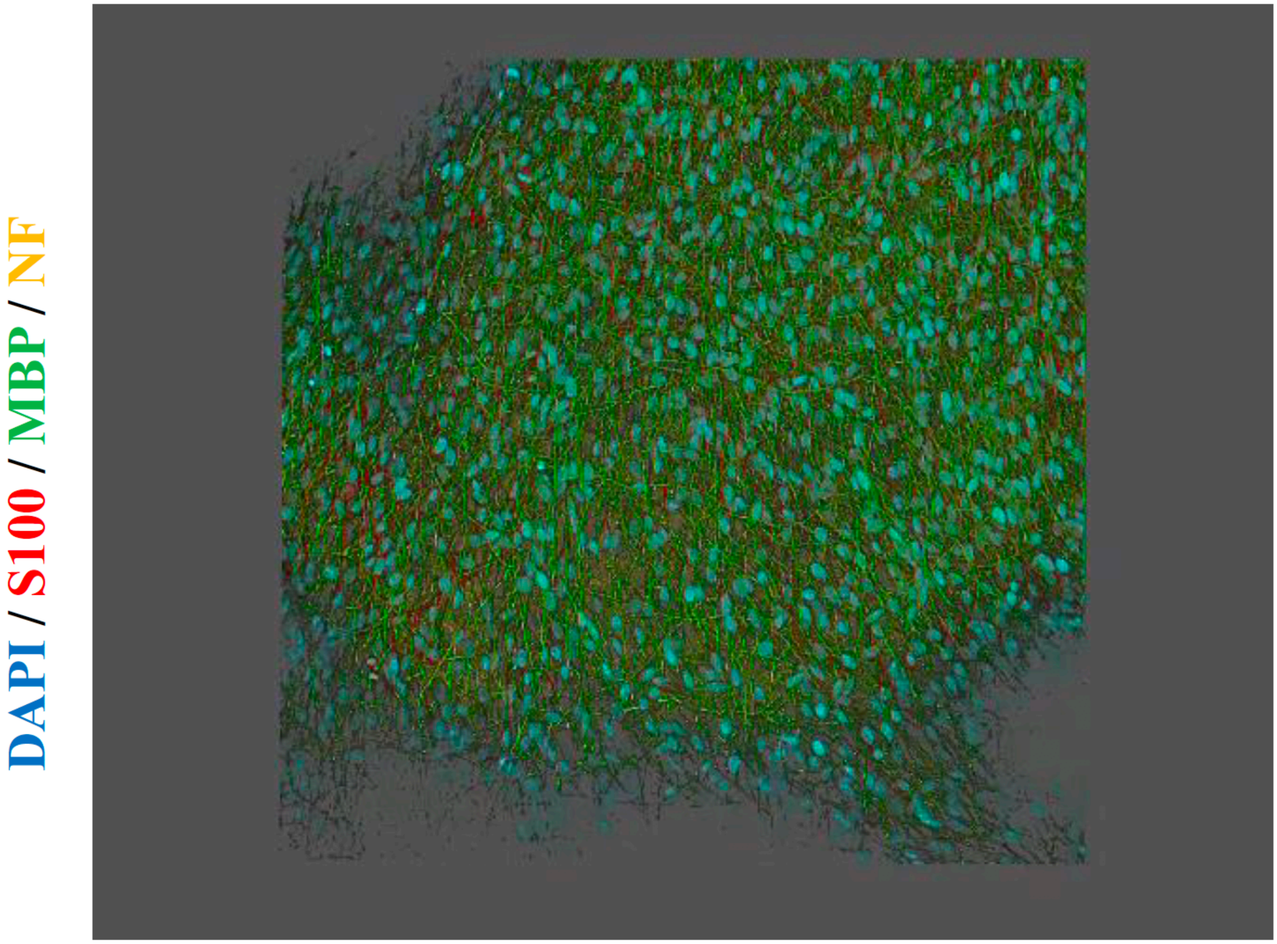

Movie 2

Diff. SC in Sol. NRG1

DAPI / NF / hNA

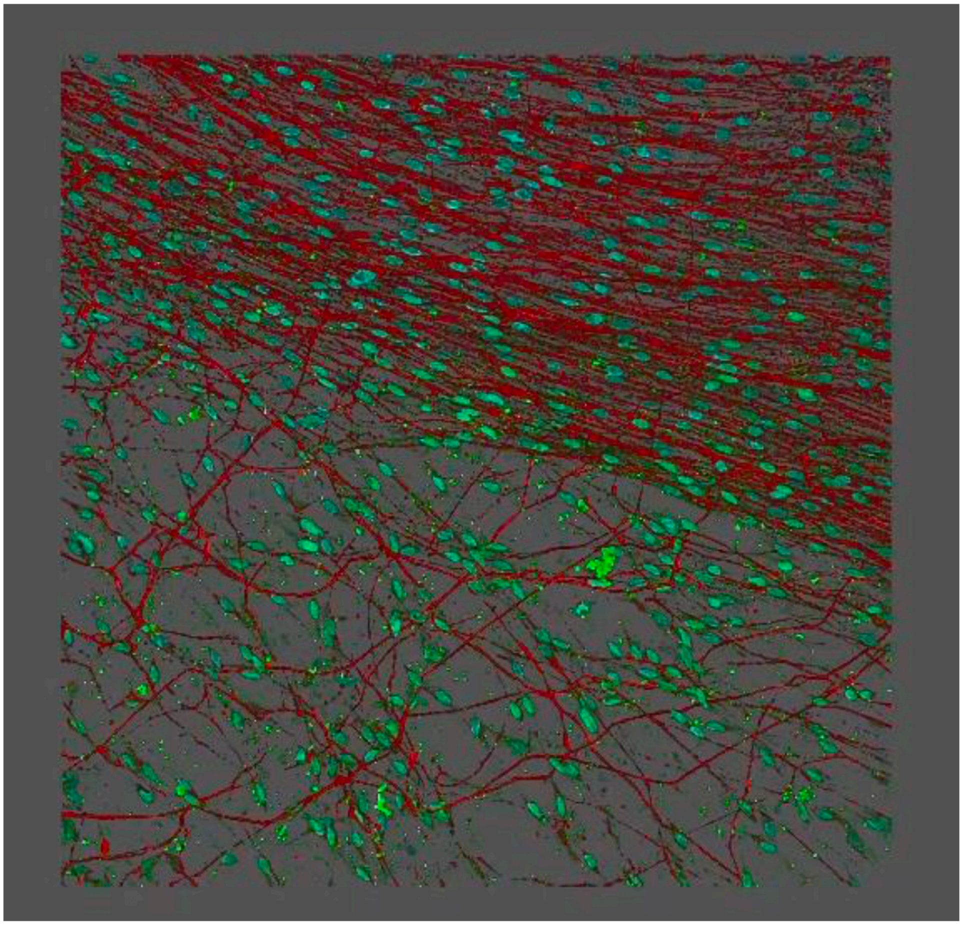

Movie 3

Diff. SC on iNRG1

DAPI / NF / hNA

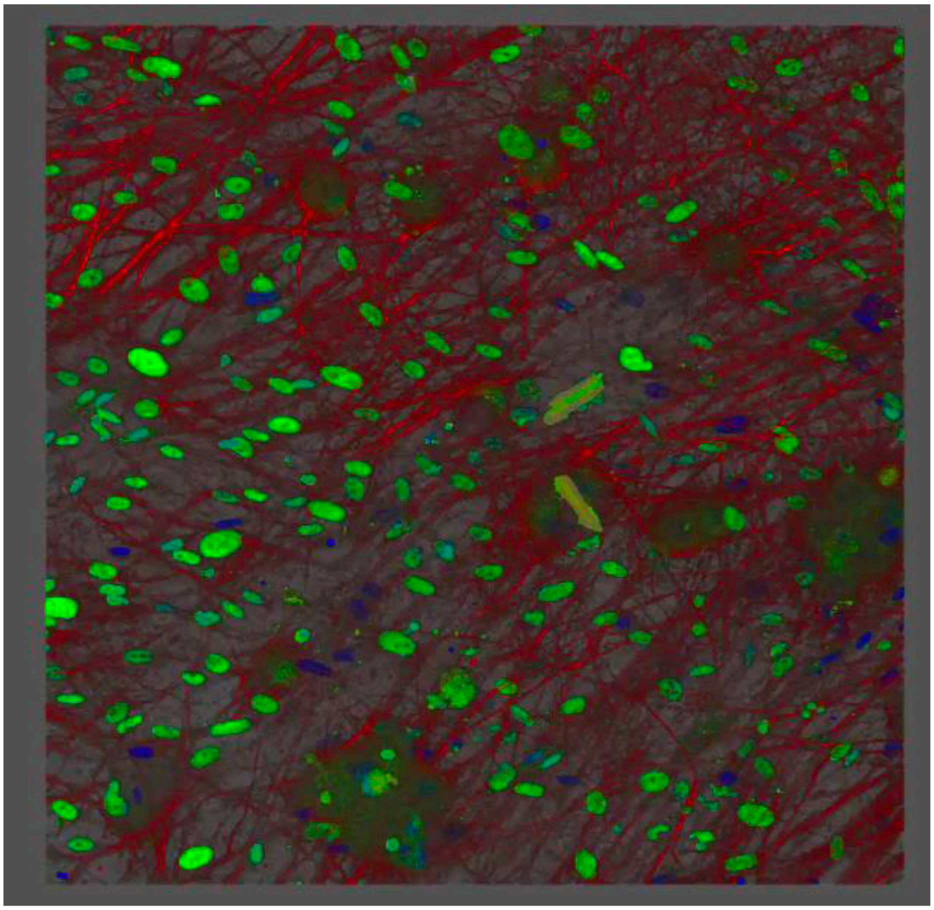

Figure S2: SC differentiated on iNRG1 localize preferentially on TUJ1+ neuronal axons. hNA+ undifferentiated KC-NC mainly localize at the embryo mesenchyme, while SC differentiated on iNRG1 migrated towards and localized close to TUJ1+ neuronal axons.

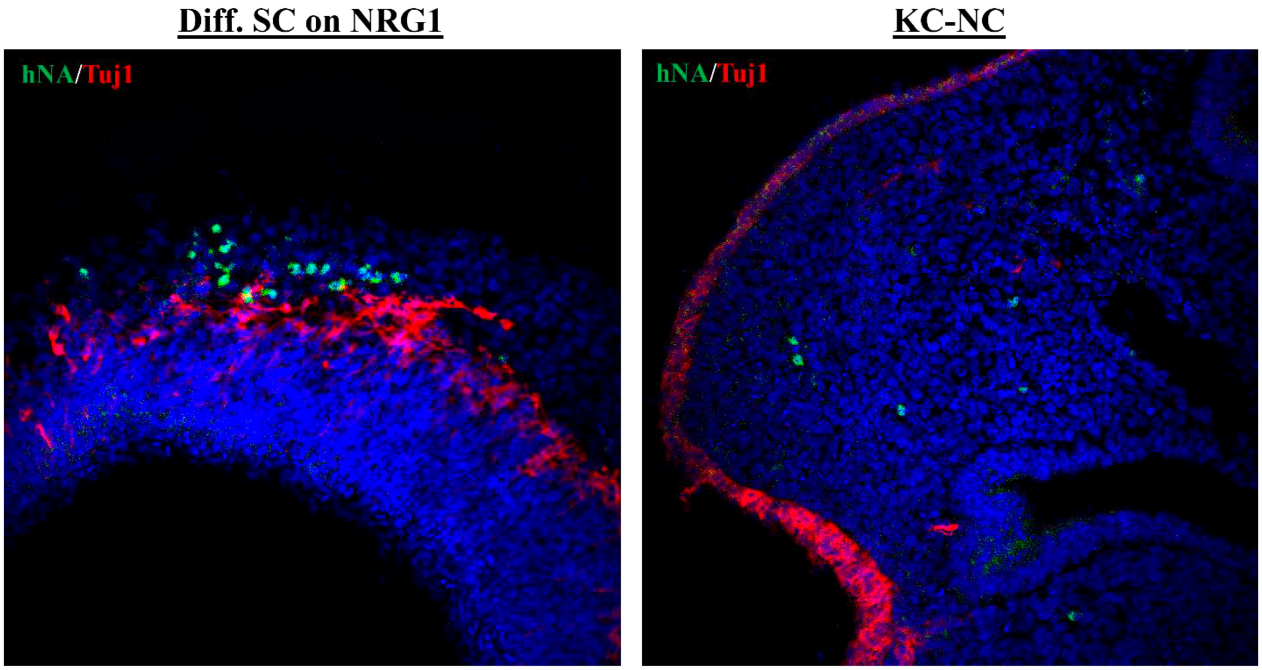

Figure S3: Single-cell RNA-seq quality control. Estimated number of cells and means reads per cell for all conditions (un-induced KC in KSFM and KC-NC in days 1, 4, 7).

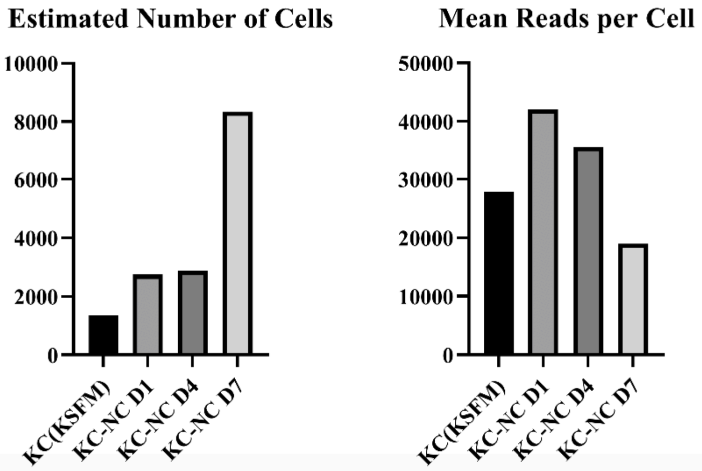

<u>Figure S4:</u> iNRG1 induces ErbB2 phosphorylation in a time and dose dependent manner. A) Western blot and quantification indicating ErbB2 phosphorylation. iNRG1 and sNRG1 concentrations were chosen in a way that the phosphorylation of ErbB2 is saturated. B) Western blot and quantification indicating that iNRG1 showed extended phosphorylation of ErbB2 for 4 hours.

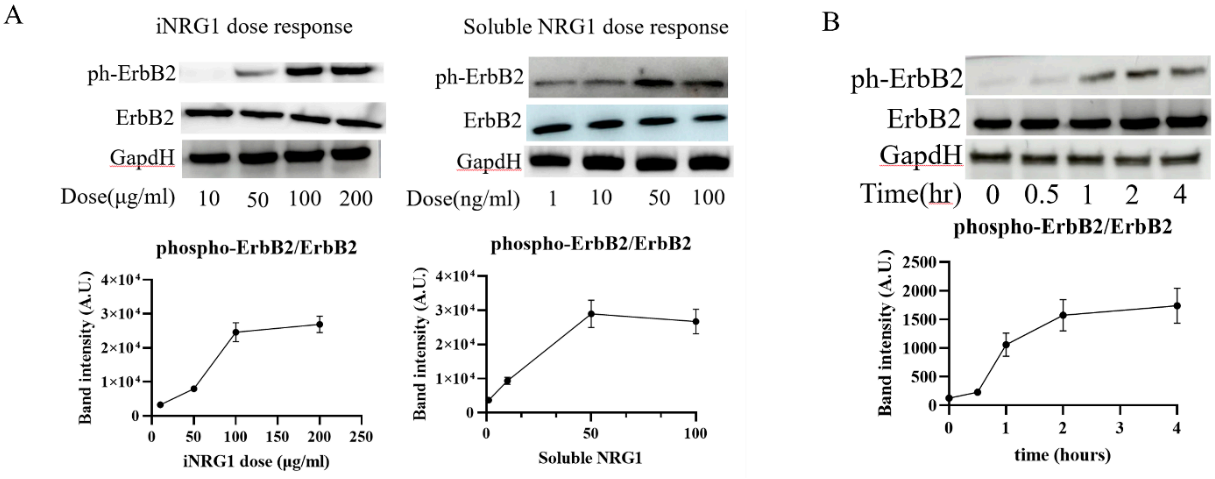

Figure S5: A) GO Pathway Enrichment Analysis of the top 25 biological processes upregulated on differentiation of SC on iNRG1 indicates that a number of them are related to Schwann cell development and signaling (highlighted in red). B) Other upregulated biological processes and cellular components are involved in PNS cells and nervous system development. C) Pathways upregulated by sNRG1 are not related to neuronal or Schwann cell function.

A

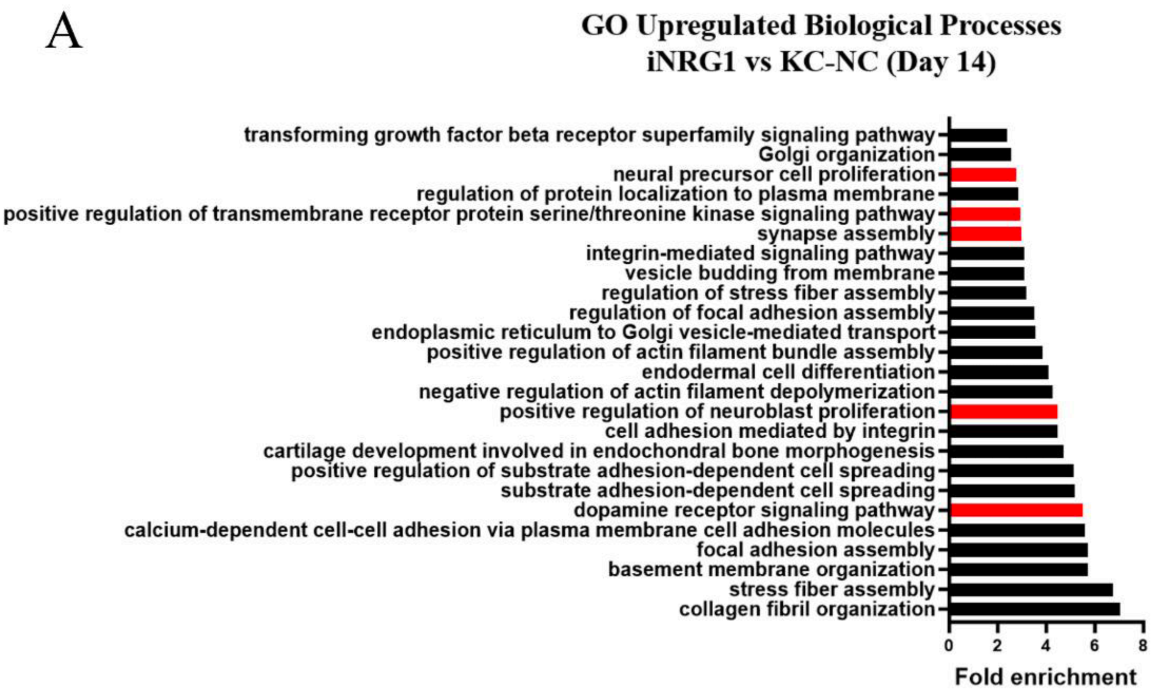

B

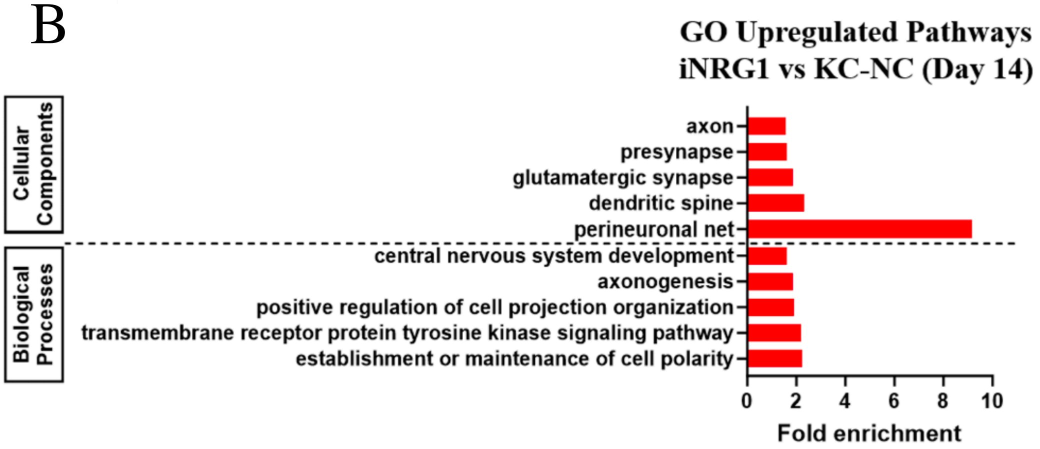

C

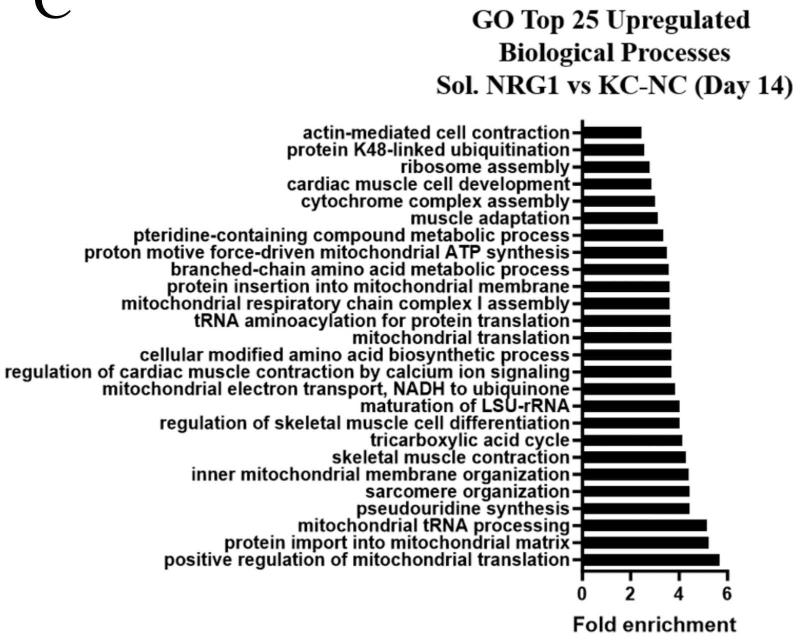

Table S1: RT-PCR primer list

Gene Name	Forward	Reverse	Gene Name	Forward	Reverse
ErbB2	TGCAGGGAAACC TGGAACTC	ACAGGGGTGGTA TTGTTCAGC	Sox2	GCCGAGTGGAAA CTTTTGTCG	GGCAGCGTGTAC TTATCCTTCT
ErbB3	GGTGATGGGGAA CCTTGAGAT	CTGTCACTTCTCG AATCCACTG	Pax3	AGCTCGGCGGTG TTTTTATCA	CTGCACAGGATCT TGGAGACG
Oct6	CGCTCTACGGTAA CGTGTTCT	CCAAGCCGGTGA TCTCGTG	SOX10	CCTCACAGATCGC CTACACC	CATATAGGAGAAG GCCGAGTAGA
GFAP	CTGCGGCTCGATC AACTCA	TCCAGCGACTCA ATCTTCCTC	PLP1	ACCTATGCCCTGA CCGTTG	TGCTGGGGAAGG CAATAGACT
VIM	GACGCCATCAAC ACCGAGTT	CTTTGTCGTTGGT TAGCTGGT	MPB	GGCCGGACCCAA GATGAAAA	CCCCAGCTAAATC TGCTCAGG
NFκB	AACAGAGAGGAT TTCGTTTCCG	TTTGACCTGAGG GTAAGACTTCT	PMEL	AGGTGCCTTTCTC CGTGAG	AGCTTCAGCCAG ATAGCCACT
MPZ	CATCGTGGTTTAC ACCGACAG	TGGAAGATCGAA ATGGCATCTCT	LATS1	AATTTGGGACGC ATCATAAAGCC	TCGTCGAGGATCT TGGTAACTC
PMP22	ATCGTCAGCCAAT GGATCGTG	AGAAACAGTGGT GGACATTTCC	YAP	TAGCCCTGCGTAG CCAGTTA	TCATGCTTAGTCC ACTGTCTGT
Krox20	TCAACATTGACAT GACTGGAGAG	AGTGAAGGTCTG GTTTCTAGGT	TAZ	CACCGTGTCCAAT CACCAGTC	TCCAACGCATCAA CTTCAGGT
Periaxin	GGTGGAAATTATC GTGGAGACG	GCAGCTCCCGAA CGAAGAT	TEAD1	ATGGAAAGGATG AGTGACTCTGC	TCCCACATGGTGG ATAGATAGC
FGF2	TCGTGGAGGACA AGTTGGTG	TTGCAGGGCTTG ATGATCCG	TEAD2	CTTCGTGGAACC GCCAGAT	GGAGGCCACCCT TTTTCTCA
c-Jun	TCCAAGTGCCGA AAAAGGAAG	CGAGTTCTGAGCT TTCAAGGT			
CDK1	AAACTACAGGTC AAGTGGTAGCC	TCCTGCATAAGCA CATCCTGA			

Table S2: Antibody List

Assay	Antibody	Catalog #	Dilution	Assay	Antibody	Catalog #	Dilution
WB	ErbB2	MA5-13105	1:1000	IHC	MBP	ab40390	1:300
WB	ErbB3	mAb#12708	1:1000	IHC	PLP1	ab105784	1:200
WB	GapdH	mAb#2118	1:10000	IHC	GFAP	ab7260	1:100
WB	pAKT	#9271	1:1000	IHC	Krox20	mAb#9749	1:200
WB	AKT	#9272	1:1000	IHC	Nestin	MAB5326	1:200
WB	pMAP38	mAb#4511	1:1000	IHC	S100b	EP1576Y	1:200
WB	Map38	mAb#8690	1:1000	IHC	PMEL	ab238048	1:200
WB	pErk1/2	#9101	1:1000	IHC	MPZ	ab31851	1:100
WB	Erk1/2	#9202	1:1000	IHC	YAP	mAb#12395	1:200
WB	pSAPK-JNK	#9251	1:1000	Histology	HNA	ab191181	1:100
WB	SAPK-JNK	#9252	1:1000	Histology	NF	MAB2216	1:100
IHC	Pax3	ab190754	1:200	Histology	YAP	mAb#14074	1:100
IHC	Sox10	mAb#89356	1:200	Histology	Tuj-1	MAB1195	1:200
